# New Insights Into the Activity of Apple Dihydrochalcone Phloretin: Disturbance of Auxin Homeostasis as Physiological Basis of Phloretin Phytotoxic Action

**DOI:** 10.3389/fpls.2022.875528

**Published:** 2022-07-07

**Authors:** Dijana Smailagić﻿﻿﻿, Nevena Banjac, Slavica Ninković﻿﻿﻿, Jelena Savić﻿﻿﻿, Tatjana Ćosić﻿﻿﻿, Aleš Pěnčík, Dušica Ćalić﻿﻿﻿, Milica Bogdanović﻿﻿﻿, Milena Trajković﻿﻿﻿, Mariana Stanišić

**Affiliations:** ^1^Institute for Biological Research “Siniša Stanković” – National Institute of Republic of Serbia, University of Belgrade, Belgrade, Serbia; ^2^Laboratory of Growth Regulators, Faculty of Science, Palacký University and Institute of Experimental Botany, The Czech Academy of Sciences, Olomouc, Czechia

**Keywords:** allelopathy, ARD, auxin, dihydrochalcones, *Malus* × *domestica* Borkh. (apple), phloretin, phytotoxicity, polar auxin transport

## Abstract

Apple species are the unique naturally rich source of dihydrochalcones, phenolic compounds with an elusive role *in planta,* but suggested auto-allelochemical features related to “apple replant disease” (ARD). Our aim was to elucidate the physiological basis of the phytotoxic action of dihydrochalcone phloretin in the model plant Arabidopsis and to promote phloretin as a new prospective eco-friendly phytotoxic compound. Phloretin treatment induced a significant dose-dependent growth retardation and severe morphological abnormalities and agravitropic behavior in Arabidopsis seedlings. Histological examination revealed a reduced starch content in the columella cells and a serious disturbance in root architecture, which resulted in the reduction in length of meristematic and elongation zones. Significantly disturbed auxin metabolome profile in roots with a particularly increased content of IAA accumulated in the lateral parts of the root apex, accompanied by changes in the expression of auxin biosynthetic and transport genes, especially *PIN1*, *PIN3*, *PIN7*, and *ABCB1*, indicates the role of auxin in physiological basis of phloretin-induced growth retardation. The results reveal a disturbance of auxin homeostasis as the main mechanism of phytotoxic action of phloretin. This mechanism makes phloretin a prospective candidate for an eco-friendly bioherbicide and paves the way for further research of phloretin role in ARD.

## Introduction

An array of plants’ secondary metabolites can adversely affect the growth and development of neighboring plants by various physiological and biochemical mechanisms in a process known as allelopathy (Weir et al., 2004; Cheng and Cheng, 2015). Allelopathic interactions predominate in natural and agricultural ecosystems, where they create different ecological and economic implications. Stunted growth of crops and low yields and crop quality due to soil sickness (Huang et al., 2013a), unsuccessful forest regeneration (Souto et al., 2000) and biological invasion of exotic plant species (Bais et al., 2003) are all the consequences of allelopathic activity. On the other hand, allelopathic interactions between crops and weeds have a promising future in strategies for cropping systems improvement (De Albuquerque et al., 2011). The allelochemicals involved in these interactions can be considered as prospective bioherbicides based on new mode-of-actions (MOA) for managing the global problem of rapidly increasing weed resistance to actual synthetic herbicides.

**Figure 1:**
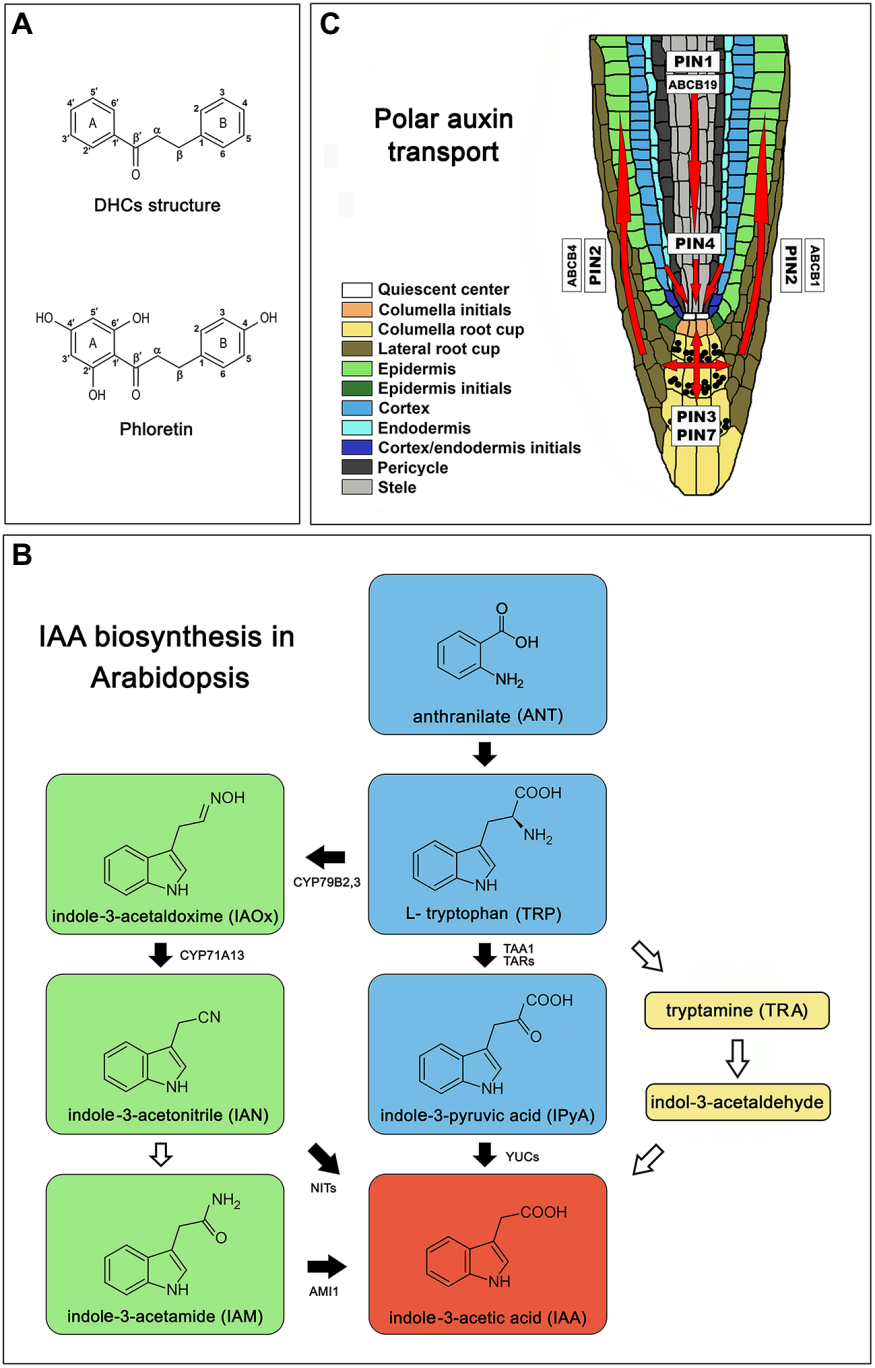
Introductory picture. **(A)** Chemical structure of dihydrochalcones (DHCs) and phloretin. **(B)** Schematic representation of IAA biosynthesis in Arabidopsis [*Arabidopsis thaliana* (L.) Heynh.] Col-0 based on a review of Brumos et al. (2014). Green color represents pathway specific to the genus *Brassica* (Sugawara et al., 2009). Black arrows indicate well characterized steps. White arrows mark poorly understood steps or incomplete pathways. **(C)** Polar auxin transport in Arabidopsis root based on Vieten et al. (2005), Michniewicz et al. (2007) and Geisler et al. (2017); red arrows indicate directions of auxin flow. AMI1 (indole-3-acetamide hydrolase), ABCB (ATP-binding cassette-B), CYP71A13 (indole-acetaldoxime dehydratase CYP71A13), CYP79B2,3 (cytochrome P450 monooxygenases CYP79B2 and CYP79B3), PIN (pin-formed), NITs (nitrilases), TAA1 (tryptophan aminotransferase of Arabidopsis 1), TAR2 (tryptophan aminotransferase related 2), YUC (flavin-binding monooxygenase).

Despite their great diversity, plant allelochemicals can be grouped into three main chemical classes: terpenoids, N-containing compounds, and phenolic compounds (Bachheti et al., 2020). Dihydrochalcones (DHCs) are a special group of phenolic compounds with limited occurrence in the plant kingdom, whose structure is closely related to flavonoid precursors, chalcones. DHCs are defined by the presence of two C6 rings joined by a C3 bridge, but the double bond is reduced in comparison with chalcones (Figure 1A). The unique naturally rich source of DHCs are species of the genus *Malus*, including the domestic apple (*Malus × domestica* Borkh.) whose DHCs make up 96–97% of the total phenolics in the leaves and represent approximately 10%–20% of DW leaf tissue (Pontais et al., 2008; Dugé de Bernoville et al., 2011). Phlorizin (phloretin 2´-O-glucoside) is the first discovered and the most common dihydrochalcone in *Malus* species. The glycosylation of phloretin [3-(4-hydroxyphenyl)-1-(2,4,6-trihydroxyphenyl)-1-propanone; Figure 1A] at position 2′ by specific uridine diphosphate (UDP)-glucose: phloretin 2′-O-glucosyltransferase is the key step in phlorizin biosynthesis that directly determines the concentration of phlorizin in apple tissues (Zhou et al., 2017). Besides phlorizin, there are at least 10 other DHCs described in the genus *Malus* that differ in sugar group and/or their binding sites, and all are derivatives of aglycone phloretin (Gosch et al., 2010). Due to its typical DHCs structure (Figure 1A), phloretin is a very flexible molecule which can react efficiently with biological macromolecules. The reactivity of phloretin results in its striking health-protecting properties in mammalians and humans such as antimicrobial, anticancer, antioxidant, estrogenic, anti-inflammatory, cytoprotective, and neuroprotective activity (Nithiya and Udayakumar, 2016; Behzad et al., 2017). However, the role of phloretin and other DHCs in plants still remains elusive. Börner (1959, 1960) was the first who suggested autotoxicity of phloretin and phlorizin and related them with “apple replant disease” (ARD), which is characterized by stunted growth, reduced root system and low yields in apple trees replanted on lands that previously supported apple orchards. High concentrations of phlorizin (1–4 mM) were phytotoxic for *Malus hupehensis* and decreased photosynthesis and respiration rate and increased malondialdehyde content and antioxidative enzymes activities (Jianghong et al., 2007). Yin et al. (2018) suggested that phlorizin and phloretin were harmful to *M. hupehensis* seedlings at concentrations found in the orchards´ soil, which led to up-regulation of genes encoding defense system-related and free radical scavenging proteins.

To facilitate the examination of phytotoxic attributes of apple root exudates and to exclude possible microbial influence, we recently developed a model system based on the apple hairy root system *in vitro* (Stanišić et al., 2019). There, we reported a significant inhibitory effect of apple hairy root exudate on the development and growth of the model species Arabidopsis. In the apple hairy root growth medium, in addition to chlorogenic and caffeic acids, dihydrochalcones phlorizin and its aglycone phloretin were detected. Since the determination of structure/activity showed that the presence of a free hydroxyl group at the position C2ʹ and the absence of glycosyl molecules allow phloretin to react more intensively with biological molecules compared to phlorizin (Barreca et al., 2014), we hypothesized that phloretin contributed much more to the observed phytotoxicity of apple root exudate in Arabidopsis than phlorizin.

Phenolic compounds have been frequently reported to affect plant development by modulating the homeostasis of plant hormone auxin (Brown et al., 2001; Peer and Murphy, 2007; Ahmed et al., 2020). In apple, silencing of chalcone synthase CHS led to the loss of almost all flavonoid and dihydrochalcone compounds, and induced significant phenotypic effects, which included a highly dwarfed growth pattern presumably as a result of significantly increased auxin transport from shoots to root (Dare et al., 2013). Downregulation of phloretin-specific glycosyltransferase UGT88F1 led to decrease in the concentration of phlorizin, phloretin and a number of other polyphenolic compounds in transgenic apple plants. These changes increased auxin flux from the shoot apex and produced a highly dwarfed phenotype that phenocopied previously characterized CHS-silenced plants (Dare et al., 2017).

Auxin plays a fundamental role in plant development and coordinates plant responses to the constantly changing environment through the regulation of cell division, elongation and differentiation of the cells in meristematic tissues. Delicately regulated co-action of auxin biosynthesis, conjugation, degradation and transport through plant tissues generates morphogenic gradients of auxin that govern cell fate decisions and underlies plant phenotypic plasticity. The biologically active native plant auxin indole-3-acetic acid (IAA) is biosynthesized in Arabidopsis mainly *via* a tryptophan-dependent metabolic pathway containing four biosynthetic routes: the indole-3-pyruvic acid (IPyA), the indole-3-acetaldoxime (IAOx), indole-3-acetamide (IAM), and tryptamine (TRA) route (Normanly et al., 1993; Figure 1B). The majority of IAA is produced *via* the IPyA metabolic pathway (Mashiguchi et al., 2011; Stepanova et al., 2011; Won et al., 2011) where TRYPTOPHAN AMINOTRANSFERASE OF ARABIDOPSIS 1/TRYPTOPHAN AMINOTRANSFERASE RELATED (TAA1/TAR) catalyzes the conversion of tryptophan to IPyA (Stepanova et al., 2008; Won et al., 2011) and the YUCCA (YUC) family of flavin monooxygenases (YUC1 – YUC11) further converts IPyA to IAA (Brumos et al., 2014). The IAOx route is largely restricted to the genus *Brassica* (Sugawara et al., 2009) where cytochrome P450 monooxygenases: CYP79B2 and CYP79B3 catalyze the conversion of Trp to IAOx which can be further converted to indole-3-acetamide (IAM) and indole-3-acetonitrile (IAN), both capable to produce IAA (Zhao et al., 2002; Sugawara et al., 2009). Due to the delicacy in the regulation of processes depending on auxin levels, plants have developed several mechanisms to control the levels of active auxin. One of them is the formation of inactive conjugates with amino acids, sugars or peptides that function in IAA storage or as intermediates in successive degrading steps leading to the production of irreversibly degradable oxindole-3-acetic acid (oxIAA; Hayashi et al., 2021).

Polar auxin transport (PAT) plays a central role in auxin maxima generation. PAT is mediated by auxin influx carriers, AUXIN1/LIKE AUXIN1 (AUX1/LAX), auxin efflux proteins PIN-FORMED (PIN1, PIN2, PIN3, PIN4, PIN7; Vieten et al., 2005; Michniewicz et al., 2007), as well as proteins from MULTI-DRUG RESISTANCE/P-GLYCOPROTEIN (MDR/PGP) subfamily, belonging to the ATP-BINDING CASSETTE (ABC) transporter superfamily (ABCB1, ABCB4, ABCB19; Zažímalová et al., 2010; Geisler et al., 2017). AUX1/LAX proteins are responsible for the auxin intake (Swarup et al., 2001), while PINs show asymmetric localization on cell membranes in certain cell types, correlated with known acropetal and basipetal directions of auxin flow (Vieten et al., 2005; Michniewicz et al., 2007; Figure 1C). All auxin carriers act synergistically mediating the plasticity of the root system architecture when they adapt to various environmental stimuli (Sun et al., 2010; Yuan et al., 2013; Li et al., 2015; Yuan and Huang, 2016; Bahmani et al., 2020) or respond to soil allelochemicals (Zhang et al., 2018; Li et al., 2019).

The presented study aims to evaluate DHC phloretin of apple tree as a new phytotoxic compound, addressing its basic mechanism of action, which we assume involves the modulation of auxin homeostasis. For that purpose, we investigated the growth and gravitropic response of the model plant Arabidopsis to exogenous phloretin. The obtained morphological data revealed a significant dose-dependent growth retardation and the presence of morphological abnormalities and agravitropic behavior in seedlings, especially roots. Microscopic, molecular and biochemical approaches have confirmed that roots are the primary targets of phloretin phytotoxic action. Significantly disturbed auxin metabolome profile in the root with a particularly increased IAA content and its accumulation in the lateral parts of the root apex, with noticeable changes in auxin biosynthetic and PAT-involved genes expression, confirmed the hypothesis that disturbance in auxin homeostasis is the basic mechanism of phytotoxic action of phloretin. This mechanism makes phloretin a prospective candidate for an eco-friendly bioherbicide and also paves the way for further research into the role of phloretin in ARD.

## Materials and Methods

### Plant Material

The seeds of Arabidopsis [*Arabidopsis thaliana* (L.) Heynh.] ecotype Columbia (Col-0) used in the experiments were obtained from plants grown in a glasshouse of The Institute for Biological Research “Siniša Stanković” - National Institute of Republic of Serbia, University of Belgrade. Seeds of Arabidopsis transgenic line DR5rev::GFP (NASC ID: N9361, background Col-0) were obtained from Nottingham Arabidopsis Stock Centre (NASC).

### Growth Conditions and Phloretin Treatments

Arabidopsis seeds were surface sterilized for 60 s with 96% (v/v) ethanol and commercial bleach (4%–6% NaOCl) in 1:1 ratio and placed in Petri dishes with 15 ml of solid ½MS medium (half-concentrated (Murashige and Skoog, 1962) mineral salts and (Linsmaier and Skoog, 1965) vitamins, 3% (w/v) sucrose, 100 mg l^−1^ myo-inositol and 0.8% (w/v) agar, pH 5.8). The ½MS medium was supplemented with phloretin (Phl; Sigma, St. Louis, MO, United States) at increasing concentrations (0, 125, 250, 500, 750, 1,000 and 1,500 μM). Phloretin stock solution was freshly prepared in dimethyl-sulfoxide (DMSO; Duchefa Biochemie, Haarlem, Netherlands) and added to autoclaved and cooled ½MS medium. The final DMSO concentration in the control and phloretin-enriched media was 0.1% (v/v). After cold stratification at 4°C for 3 days in the dark, Petri dishes were transferred to light (35 ± 2 μmol m^−2^ s^−1^; 16 h: 8 h, light: dark) and kept vertically in racks at 25 ± 2°C. Petri dishes were not sealed with Parafilm to prevent excessive ethylene accumulation. The bioassay was repeated three times using a randomized design, with 10 seeds in three replicates (Petri dishes) for each treatment (*n* = 90).

To quantify Arabidopsis seed germination, seedlings growth parameters and the appearance of aberrant phenotypes, Petri dishes were inspected under a stereo microscope (Carl Zeiss, Jena, Germany) at 5, 10 and 15 days after germination (DAG) and photographed with Nikon Coolpix 4500 digital camera. The number of germinated seeds and seedlings with deformities were determined, and the percentages (%) were calculated as the number of germinated seeds or seedlings with deformities/total seed number × 100. The lengths of the primary root and the longest true leaf were measured using ImageJ 1.53e software, while the number of lateral and adventitious roots was counted under the stereo microscope. Percentages of inhibition (%) of root length, leaf length and lateral root number were calculated using the following formula: [1−(values the Phl-enriched medium/mean value on the control medium)] × 100. The percentages of adventitious roots (%) were calculated as the number of plants developing adventitious roots/total number of plants × 100. The number of true leaves was determined at 15 DAG. Gravitropic index measurements were performed on 5 and 10 DAG-old seedlings. ImageJ 1.53e software was used to measure root vertical gravitropic index (VGI), which was calculated for each root as a ratio of Ly:L, where Ly is the vertical distance from the root base to the tip, or the real depth of root tip penetration, and L is the root length, as described in Grabov et al. (2005).

Data from 5, 10 and 15 DAG measurements were analyzed separately using ANOVA. Differences between means were evaluated by Fisher’s Least Significant Difference (LSD) test at *p* < 0.05. Data was analyzed by SAS software (SAS Institute, 2002; SAS/STAT, ver. 9.00, SAS Institute Inc., Cary, NC, United States).

### Light Microscopy

The native root samples from 5 and 10 DAG-old seedlings grown vertically on the control or 500 μM phloretin-enriched medium were observed and photographed under the light Axiovert microscope (Carl Zeiss, Jena, Germany). Root cap, meristematic and elongation zone lengths were measured in 5 DAG-old seedlings using ImageJ 1.53e software. Sample roots were collected from two independent experiments (10 seedlings per experiment). Differences between mean values of the root cap, meristematic and elongation zone lengths in treatment and control were evaluated by Student’s *t*-test.

### Starch Staining

Roots from 10 DAG-old seedlings grown vertically on a control or 500 μM phloretin-enriched medium were treated with Lugol’s solution (0.37% iodine, 0.71% potassium iodide) and chloral hydrate solution (80 g chloral hydrate, 20 ml glycerol, 20 ml water) successively for 2 min and then rinsed in water. Root samples were observed under the light Axiovert microscope.

### Auxin Metabolome Profiling

Arabidopsis seeds were grown in Erlenmeyer flasks with 25 ml of sterile liquid ½MS media on an orbital shaker (95 rpm) at 16 h:8 h, light:dark. Material for analyses was obtained from 5 DAG-old seedlings treated with 500 μM phloretin (treatment) or DMSO (control) for 2 and 6 h. Each sample consisted of pooled shoots or roots of 200 seedlings which were immediately frozen in liquid nitrogen and stored at −80°C until use. The samples represented biological replicates from three independent experiments.

Quantification of auxin metabolites was performed using in-tip μSPE and ultra-fast LC–MS/MS analysis according to the method described by Pěnčík et al. (2018). Briefly, 10 mg of liquid nitrogen frozen plant material was freeze-dried and extracted with 1 ml of 50 mM phosphate buffer (pH 7.0) containing 0.1% (w/v) sodium diethyldithiocarbamate. A mixture of stable isotope-labeled auxin metabolites was added as an internal standard. A portion of 200 μl of each extract was acidified with 1 M HCl to pH 2.7 and purified by in-tip micro solid phase extraction (in-tip μSPE). For quantification of indole-3-pyruvic acid (IPyA), the another 200 μl of the extract was derivatized by cysteamine (0.75 M, pH 8.2) for 15 min, acidified with 3 M HCl to pH 2.7 and purified in-tip μSPE. After evaporation under reduced pressure, the samples were analyzed using HPLC system 1,260 Infinity II (Agilent Technologies, United States) equipped with Kinetex C18 (50 mm × 2.1 mm, 1.7 μm; Phenomenex). The LC system was linked to a 6495 Triple Quad detector (Agilent Technologies, United States).

The effect of treatment (increase or decrease of auxin metabolite content) was calculated using the following formula: Effect (%) = [(content on the Phl-enriched medium/content on the control medium) −1] × 100. Negative (−) /positive (+) values represent the percentage of auxin metabolite content increase (+) or decrease (−). Differences between the means of auxin metabolites content in treatment and control were evaluated by Student’s *t*-test.

### Gene Expression Profiling

Gene expression was measured using quantitative real-time PCR (RT-qPCR) method. Plant material was obtained from 5 DAG-old seedlings cultivated in liquid medium and treated with 500 μM phloretin (treatment) or DMSO (control) for 2, 6, 12 and 24 h. Each sample consisted of pooled shoots or roots of 400 seedlings. The samples represented biological replicates from three independent experiments.

Total RNA was extracted according to the protocol of Gasic et al. (2004) and traces of DNA were removed using DNase I (Thermo Scientific, Waltham, MA, United States). RevertAid™ Reverse Transcription Kit (Thermo Scientific) was used for reverse transcription of 400 ng of total RNA to cDNA in a 20 μl reaction. Real-time PCR analyses were carried out using QuantStudio 3 Real-Time PCR System (Applied Biosystems) and Maxima SYBR Green/ROX Kit (Thermo Scientific) with a final reaction volume of 10 μl with 10 ng of total RNA. The specific primers were designed for *TAA1*, *TAR2*, *YUC3*, *YUC4*, *YUC6*, *YUC8*, *PIN1*, *PIN2*, *PIN3*, *PIN7*, *AUX1*, *LAX3*, *ABCB1*, *ABCB4* and *ABCD19* genes (Supplementary Table S1). Protocol for qPCR consisted of: initial denaturation at 95°C for 5 min; 40 cycles of denaturation at 95°C for 30 s, annealing at 60°C for 1 min and extension at 72°C for 1 min, and the final extension at 72°C for 10 min. Analyses of melting curves consisted of cooling the reactions to 60°C followed by the increase of temperature to 95°C with a slope of 0.1°C s^−1^, while measuring the fluorescence continuously. The results were analyzed using QuantStudio Design & Analysis Software v1.4.2 (Applied Biosystems). The expression levels of tested genes under the influence of 500 μM phloretin were normalized to the reference actin gene (*ACT7*, Supplementary Table S1) and then calculated relative to the expression in corresponding controls for each time point, according to the ΔΔCt method (Livak and Schmittgen, 2001). Relative expression levels are presented as a log_2_ transformation of fold changes. Differences between mean values of relative expression levels in treatment and control were evaluated by Student’s *t*-test.

### Confocal Microscopy

The Arabidopsis transgenic line DR5rev::GFP were used to visualize and quantify the redistribution of auxin at the root tips in the presence of phloretin. Arabidopsis Col-0 and transgenic line seeds were grown vertically on solid medium containing 500 μM phloretin (treatment) or DMSO (control). At 5 DAG, fresh seedlings were mounted on microscopic slides with distilled water under a cover-slip. Confocal microscopy was performed with Leica TCS SP5 II laser scanning confocal system coupled with Leica DMI 6000 inverted microscope (Leica Microsystems, Wetzlar, Germany) equipped with HCX PL APO CS 10.0 × 0.40 DRY UV objective. For all images, single line excitation (argon laser 488 nm) and sequential scanning with multiple channel emission were used. For GFP detection, 500–530 nm was used and the channel was assigned “pseudocolor” green. The second channel was transmission. The gain and pinhole settings for GFP detection were kept constant for comparisons (gain: 1045, offset: −30, pinhole: 78.8 μm, 1.49 AU). Images were recorded taken with 5.8x digital zoom for all root comparisons, with a line average of 5. Images were acquired and exported with Leica Application suite Advanced Fluorescence 2.7.3.9723 (Leica Microsystems, Wetzlar, Germany).

The total GFP fluorescent signal area and mean fluorescent intensity were quantified using ImageJ 1.53e software. Mean fluorescent intensity was measured as the mean gray value per the region of interest (ROI) corrected for the background signal. ROI was defined as the lateral flanks of the root tip up to the quiescent center, excluding the four columns of columella cells in the center of the root tip. The roots of Col-0 seedlings did not display GFP signal under the same imaging conditions.

Sample were collected from two independent experiments. Differences between means of total GFP fluorescent signal area or fluorescent intensity in treatment and control were evaluated by Student’s *t*-test. The treatment effect (increase in total GFP fluorescent signal area or the mean fluorescent intensity) was calculated using the following formula: Effect (%) = [(Phl treatment/control)-1] × 100.

## Results

### Phloretin Significantly Inhibits the Growth and Development of Arabidopsis Seedlings

**Figure 2:**
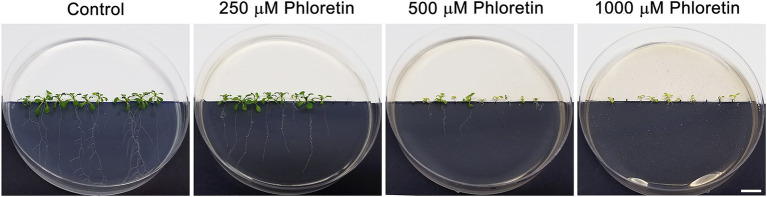
Morphology of control and phloretin-treated Arabidopsis [*Arabidopsis thaliana* (L.) Heynh.] Col-0 seedlings. Figures represent 15 days after germination (DAG)-old seedlings grown vertically on the control and phloretin-enriched medium (250, 500, and 1,000 μM). Bar, 10 mm.

**Figure 3:**
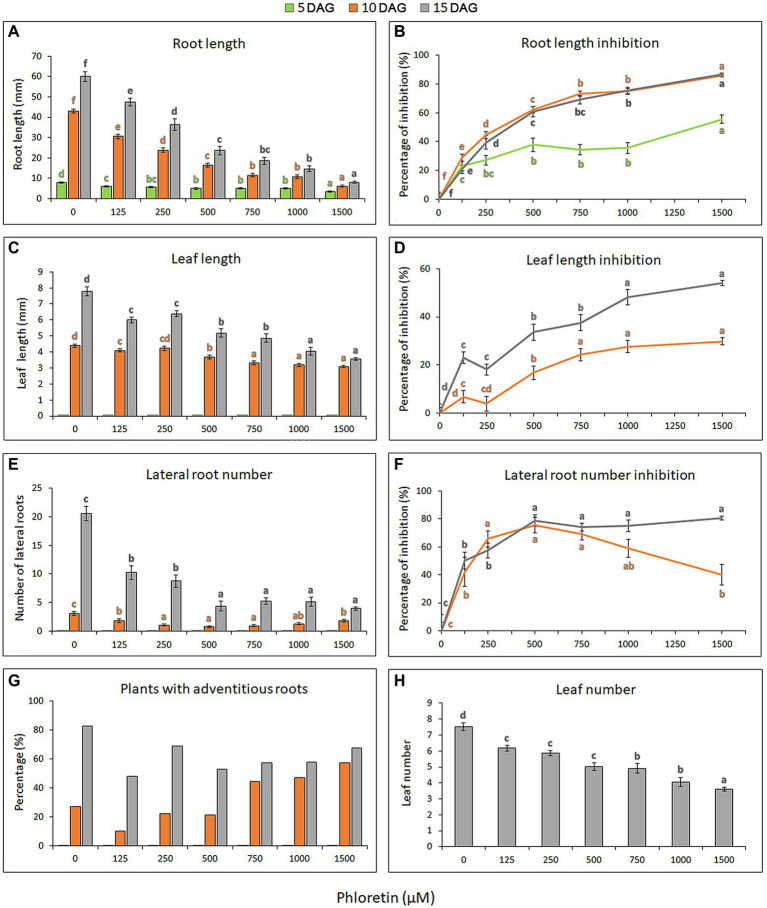
Dose-dependent effects of phloretin on Arabidopsis growth. Seeds were grown vertically on medium supplemented with phloretin in increasing concentrations (0, 125, 250, 500, 750, 1,000 and 1,500 μM). **(A)** Primary root length, **(B)** primary root length inhibition, **(C)** true leaf length, **(D)** true leaf length inhibition, **(E)** lateral root number, **(F)** lateral root number inhibition and **(G)** percentage of plants developing at least one adventitious root presented at 5, 10 and 15 d after germination (DAG); **(H)** the true leaf number presented at 15 DAG. Values represent means ± SE of 30 explants per each treatment repeated three times (*n* = 90). Values followed by different letters of the same color are significantly different at *p* < 0.05 per Fisher’s least significant difference (LSD) test.

To investigate the phytotoxic potential of phloretin, we evaluated the effects of different phloretin concentrations (0–1,500 μM) on Arabidopsis seed germination and seedlings growth and development. Phloretin had no effect on Arabidopsis seed germination at any of the tested concentrations (data not shown), but it significantly affected the morphology and growth of seedlings. The effects of phloretin were manifested primarily as the presence of morphological abnormalities and stunted growth of seedlings, which was strongly dose-dependent (Figure 2). Inhibition of primary root and true leaf growth increased with both phloretin concentration and treatment time (Figures 3A,C), and was more pronounced in root than in leaves (maximal 86.6% and 54.2% at 1,500 μM Phl at 15 DAG, respectively; Figures 3B,D). Since the true leaves at 5 DAG were just emerging, their lengths were not included in the calculations and diagrams (Figures 3C,D). Moreover, phloretin reduced lateral root development at all tested concentrations (Figure 3E). At 5 DAG, lateral roots have just emerged in both control and treated seedlings. The mid-range concentration of phloretin (250–750 μM) was most inhibitory for lateral root emergence in 10 DAG-old seedlings. Prolonged exposure to phloretin (15 DAG) reduced the number of lateral roots, especially at concentration of 500 μM and above (e.g. more than 5-fold decrease in 15 DAG-old seedlings at 1,500 μM), confirming that the inhibitory effect of phloretin increased over time (Figure 3F).

The formation of adventitious roots was also affected by phloretin. Although higher concentrations of phloretin (750–1,500 μM) could initially stimulate the emergence of adventitious roots (Figure 3G), prolonged treatment reduced their further development. Therefore, early adventitious rooting was presumably a stress-induced phenomenon.

Concentrations above 500 μM induced color change in the leaves and roots of seedlings after 10 DAG, making them slightly yellowish or brownish, respectively (Figure 2). The leaves turned into distinctly yellow or became necrotic with prolonged cultivation on phloretin (over 15 DAG; results not shown). The number of true leaves in the rosette also decreased with increasing phloretin concentration and was more than 2-fold reduced in 15 DAG-old seedlings at 1,500 μM (Figure 3H).

Overall, phloretin significantly reduced the growth of both roots and leaves of Arabidopsis seedlings in a dose-dependent manner, but with more pronounced effects on the roots. Phloretin-induced inhibition increased over time and further compromised the dynamics of Arabidopsis development.

### Phloretin Alters the Gravitropic Response of Arabidopsis Seedlings

**Figure 4:**
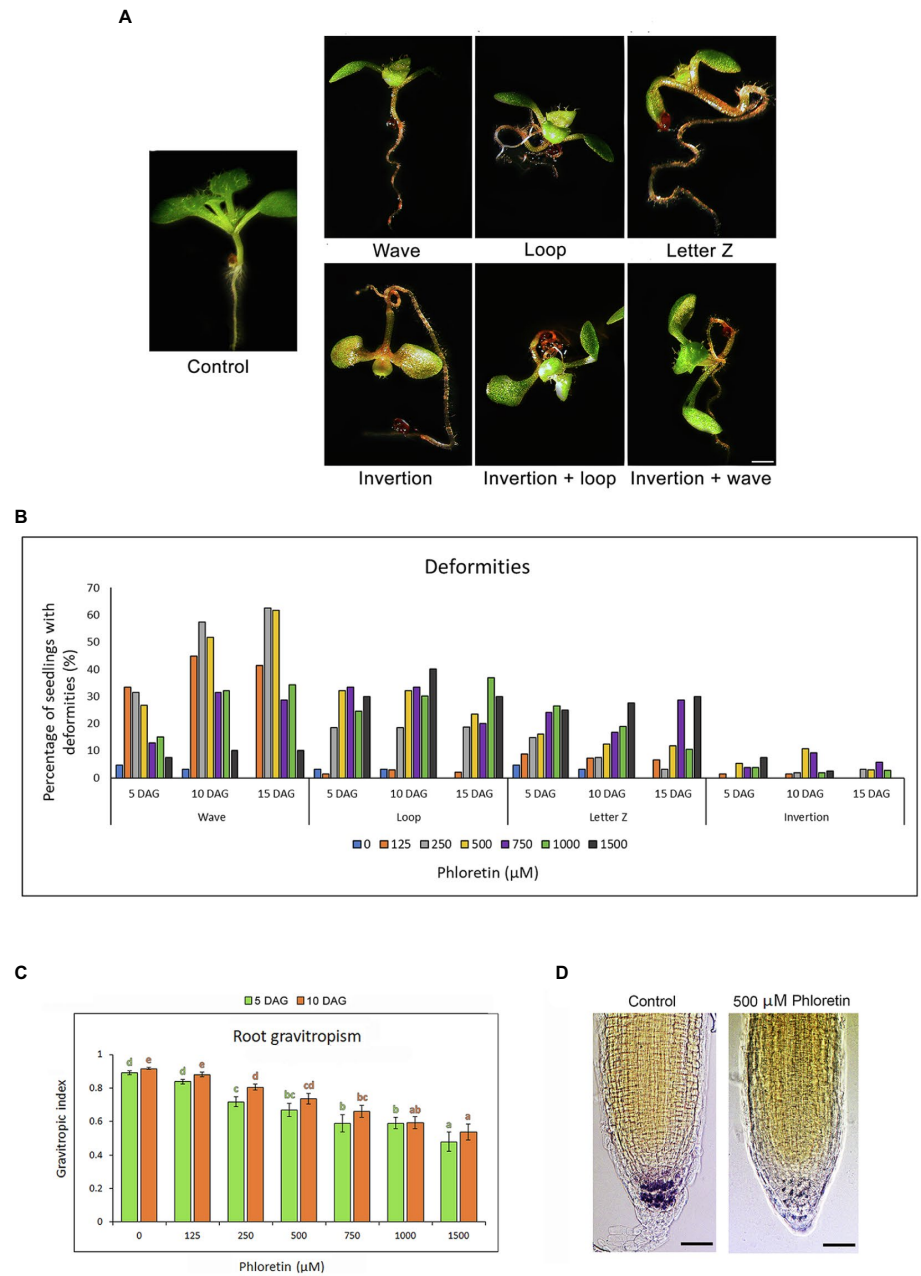
Phloretin alters the gravitropic response and induces developmental deformities in Arabidopsis. Seeds were grown vertically on medium supplemented with phloretin in increasing concentrations (0, 125, 250, 500, 750, 1,000 and 1,500 μM). **(A)** Developmental deformities in 10 d after germination (DAG)-old seedlings: wave, loop, letter Z, inversion, inversion and loop, and inversion and wave; Bar, 1 mm. **(B)** Percentage of plants with deformities at different phloretin concentrations at 5, 10 and 15 DAG. **(C)** Vertical gravitropic index (VGI) in 5 and 10 DAG-old seedlings vertically grown at different phloretin concentration. Values represents means ±SE of 30 explants per each treatment repeated three times (*n* = 90). Values followed by different letters of the same color are significantly different at *p* < 0.05 per Fisher’s least significant difference (LSD) test. **(D)** Lugol’s solution starch staining of 10 DAG-old control and 500 μM phloretin treated seedlings grown vertically. The starch in columella is colored dark purple. The representative images are shown out of three independent experiments with 10 seedlings for each experiment. Bar, 50 μm.

Phloretin significantly altered the normal development and gravitropic response of Arabidopsis seedlings, which caused the occurrence of deformities in the treated seedlings. We noticed the appearance of the four most common types of deformities according to the shape of the roots and hypocotyl, as well as the position of the epicotyl. We classified them as: wave, loop, letter Z and inversion (Figure 4A). Wave-shaped seedlings have straight hypocotyls and epicotyls with positive gravitropic roots with a large number of small waves. Seedlings in the shape of a loop and letter Z have hypocotyls and roots twisted, forming the loop or the letter Z, respectively. Sometimes, hypocotyl, cotyledons, and epicotyl grew opposite to gravity vector causing inversions (inverted growth) of the whole seedlings. Some seedlings developed two types of deformities at the same time, for example, inversion and loop or inversion and wave. The frequency of deformities depended on the duration of treatment and the concentration of phloretin (Figure 4B). The mid-range concentrations, particularly 500 μM, induced the highest number of abnormalities. The waves were dominant at lower concentrations (125–500 μM), but were also found to a lesser extent at higher ones. Some deformities have turned into others over time. Sometimes the inversions turned into loops and the letter Z shape to waves. The vertical gravitropic root index (VGI) was significantly decreased in both 5 and 10 DAG-old seedlings in all phloretin treatments except for 125 μM phloretin (Figure 4C), apparently confirming a strong decrease in the gravitropic root response.

Since the starch-filled amyloplasts in root cap columella cells play an important role in the initial steps of gravitropic stimulus perception (Baldwin et al., 2013), the effect of 500 μM phloretin on amyloplasts at the root tips of Arabidopsis seedling was evaluated at 10 DAG. Phloretin significantly reduced the starch content in the treated seedlings (Figure 4D), presumably affecting the gravity sensing process of roots.

### Arabidopsis Root Architecture Is Profoundly Disturbed by Phloretin

**Figure 5:**
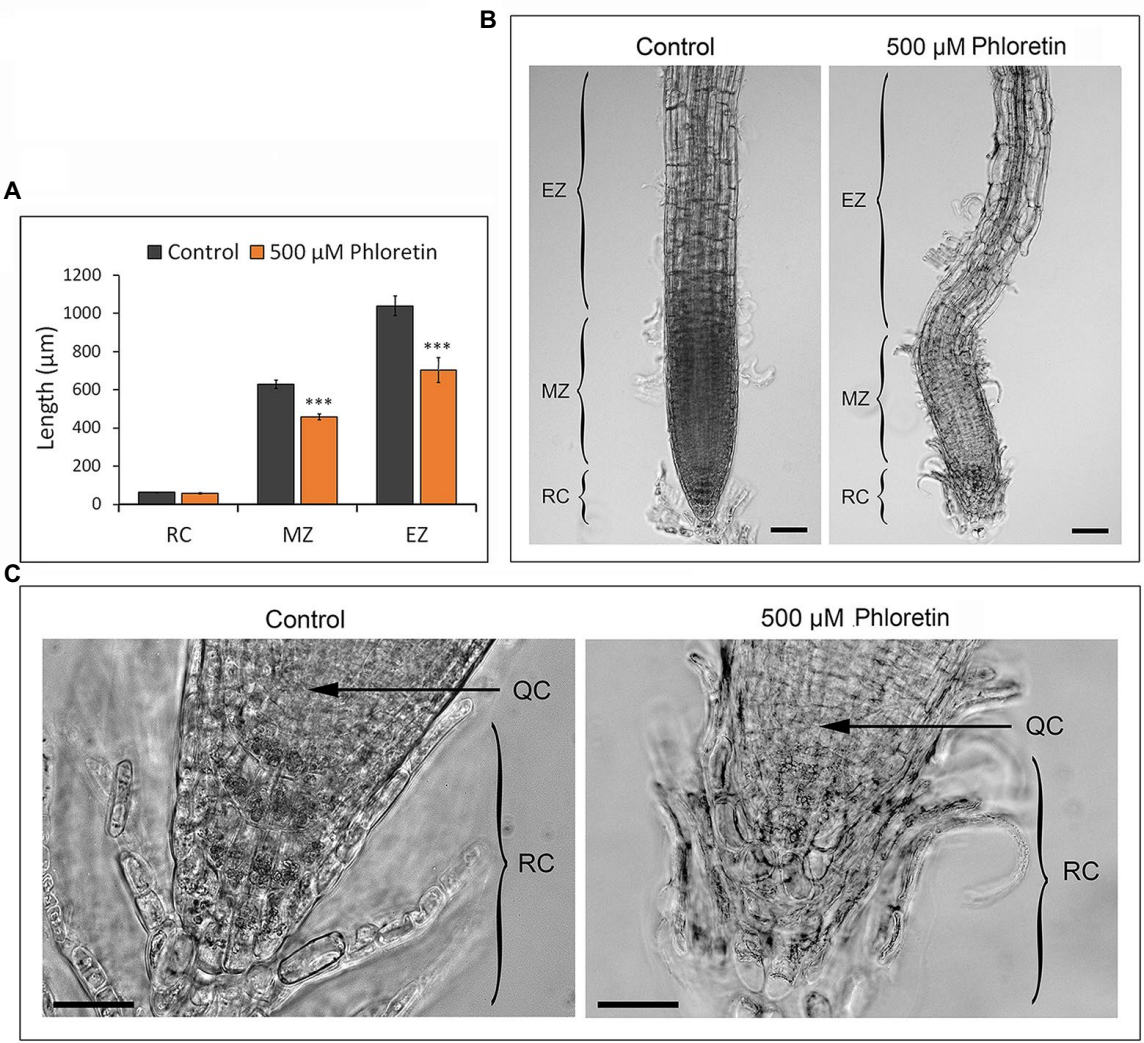
Phloretin reduces the length of the root meristematic and elongation zones and disturbs root architecture in Arabidopsis. **(A)** Length of root cap, meristematic and elongation zone of 5 d after germination (DAG)-old seedlings vertically grown on control or 500 μM phloretin-enriched medium. Values are presented as means ±SE of 20 seedlings (10 from each of two independent experiments). Asterisks indicate statistically significant difference at ***, *p* < 0.001 based on Student’s *t*-test. **(B)** Root architecture; Bar, 50 μm, and **(C)** root apex architecture of 10 DAG-old seedlings vertically grown on control or 500 μM phloretin-enriched medium; Bar, 25 μm. Arrows indicate QC. Elongation zone (EZ); meristematic zone (MZ); root cap (RC); quiescent center (QC). The representative images are shown out of two independent experiments.

We analyzed the root architecture of 5 and 10 DAG-old Arabidopsis seedlings grown vertically on control or 500 μM phloretin-enriched medium. The length of meristematic and elongation zones was significantly reduced on phloretin treatment (Figure 5A). The epidermal and cortex cells of the elongation zone were hypertrophied and shortened compared to control. Sporadically, epidermal cells separated from the subjacent cell layers, especially in the meristematic and elongation zone (Figure 5B). Columella cells in apical layers of the root cap in phloretin treated seedlings were more rounded, disorganized and loose compared to the control. The cells in the basal layers of columella, closer to the quiescent center, were often irregularly shaped and shorter than the corresponding cells in the control seedlings (Figure 5C). The quiescent center was often barely recognizable. However, the length of the root cap in control and phloretin-treated seedlings did not differ significantly (Figure 5A).

### Phloretin Significantly Disturbs the Auxin Metabolome Profile in Roots

**Figure 6:**
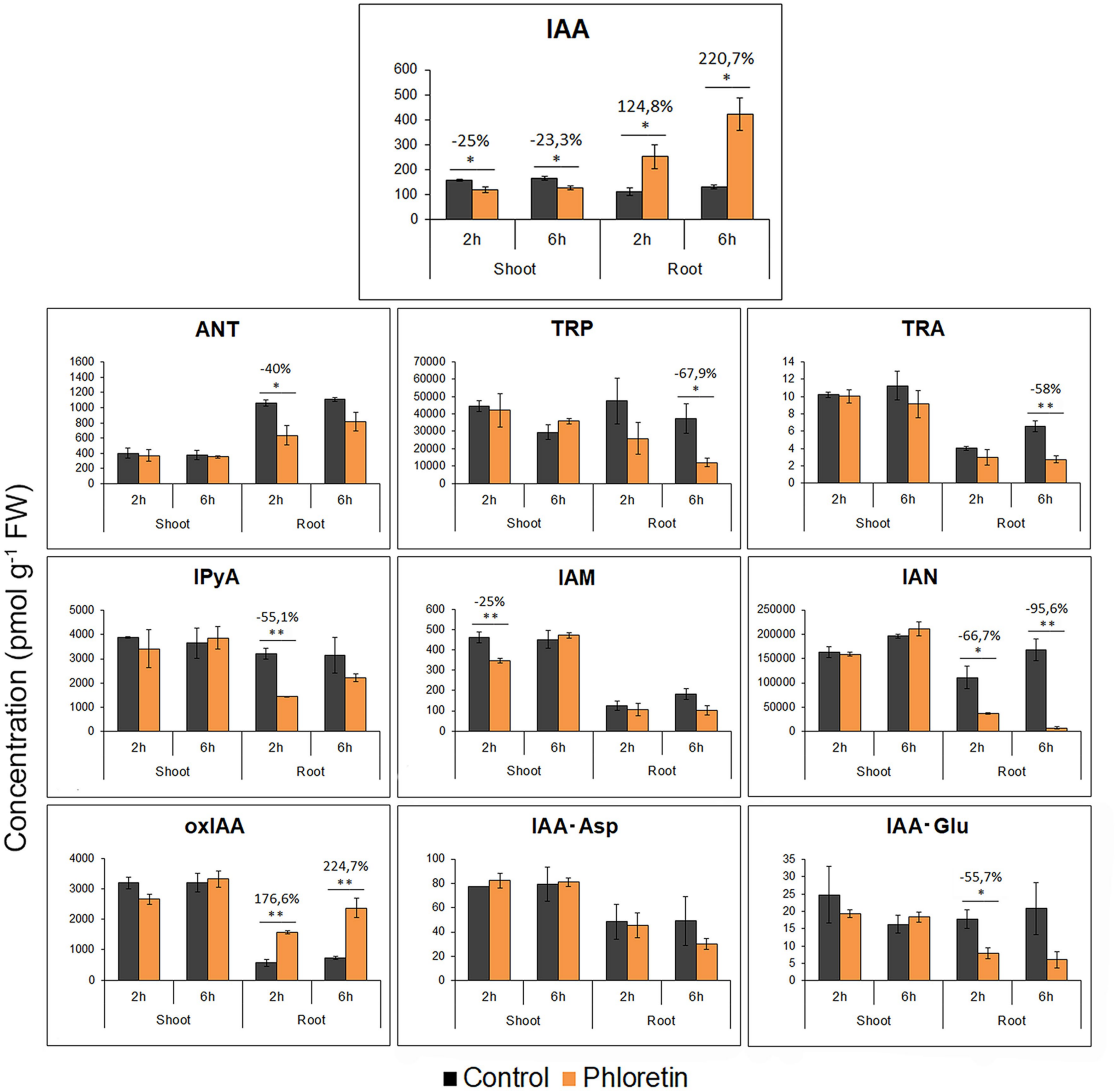
Phloretin disturbs the auxin metabolome profile in Arabidopsis. Quantification of IAA (Indole-3-acetic acid), IAA precursors: anthranilate (ANT), tryptophan (TRP), tryptamine (TRA), indole-3-pyruvic acid (IPyA), indole-3-acetamide (IAM), indole-3-acetonitrile (IAN); IAA degradation intermediate: 2-oxindole-3-acetic acid (oxIAA); IAA conjugates: indole-3-acetylaspartic acid (IAA-Asp) and indole-3-acetylglutamic acid (IAA-Glu) in 5 days after germination (DAG)-old control seedlings or seedlings treated with 500 μM phloretin for 2 or 6 h. Values are presented as means ± SE of three biological replicates (samples from three independent experiments). Asterisks indicate statistically significant difference at *p < 0.05 and **p < 0.01 based on Student’s *t*-test, while numbers above the bars indicate the percentage of auxin metabolite content increase (+) or decrease (−) compared to control.

To test the hypothesis of altered auxin homeostasis as a basis for growth retardation and agravitropic phenotype of phloretin-treated Arabidopsis seedlings, we analyzed the content of IAA and 9 prevalent IAA metabolites in shoots and roots of 5 DAG-old control seedlings and seedlings treated with 500 μM phloretin for 2 and 6 h (Figure 6).

In roots, auxin metabolome profile was significantly disturbed due to phloretin treatment. IAA levels increased rapidly after phloretin application (2.2- and 3.2-fold at 2 and 6 h of treatments), while the content of key auxin biosynthetic precursors: anthranilate (ANT), tryptophan (TRP), tryptamine (TRA), indole-3-pyruvic acid (IPyA) and indole-3-acetonitrile (IAN) were reduced. The content of 2-oxindole-3-acetic acid (oxIAA) in Arabidopsis roots was significantly elevated and closely followed the increase of IAA levels at both 2 and 6 h of treatment, while the quantity of IAA conjugates, indole-3-acetylaspartic acid (IAA-Asp) and indole-3-acetylglutamic acid (IAA-Glu) was not significantly altered or was lower relative to control (Figure 6). Presumably, in response to elevated IAA levels, phloretin intensified the degradation of IAA to oxIAA by the rapid conversion of IAA-Glu and IAA-Asp.

In shoots, phloretin induced a small but statistically significant decrease in IAA content (Figure 6). However, these small changes in IAA levels did not affect the quantity of auxin metabolites, except for IAM, whose level decreased by 25% after 2 h of treatment.

### Phloretin Induces Changes in the Expression of Genes Involved in Auxin Biosynthesis and Polar Auxin Transport

Significantly elevated IAA levels in roots shortly after application of phloretin could be the result of altered expression of genes involved in auxin biosynthesis and/or PAT. To test this assumption, RT-qPCR was used to quantify the relative expressions of key genes involved in the major auxin biosynthesis pathway (IPyA rout) and PAT.

**Figure 7:**
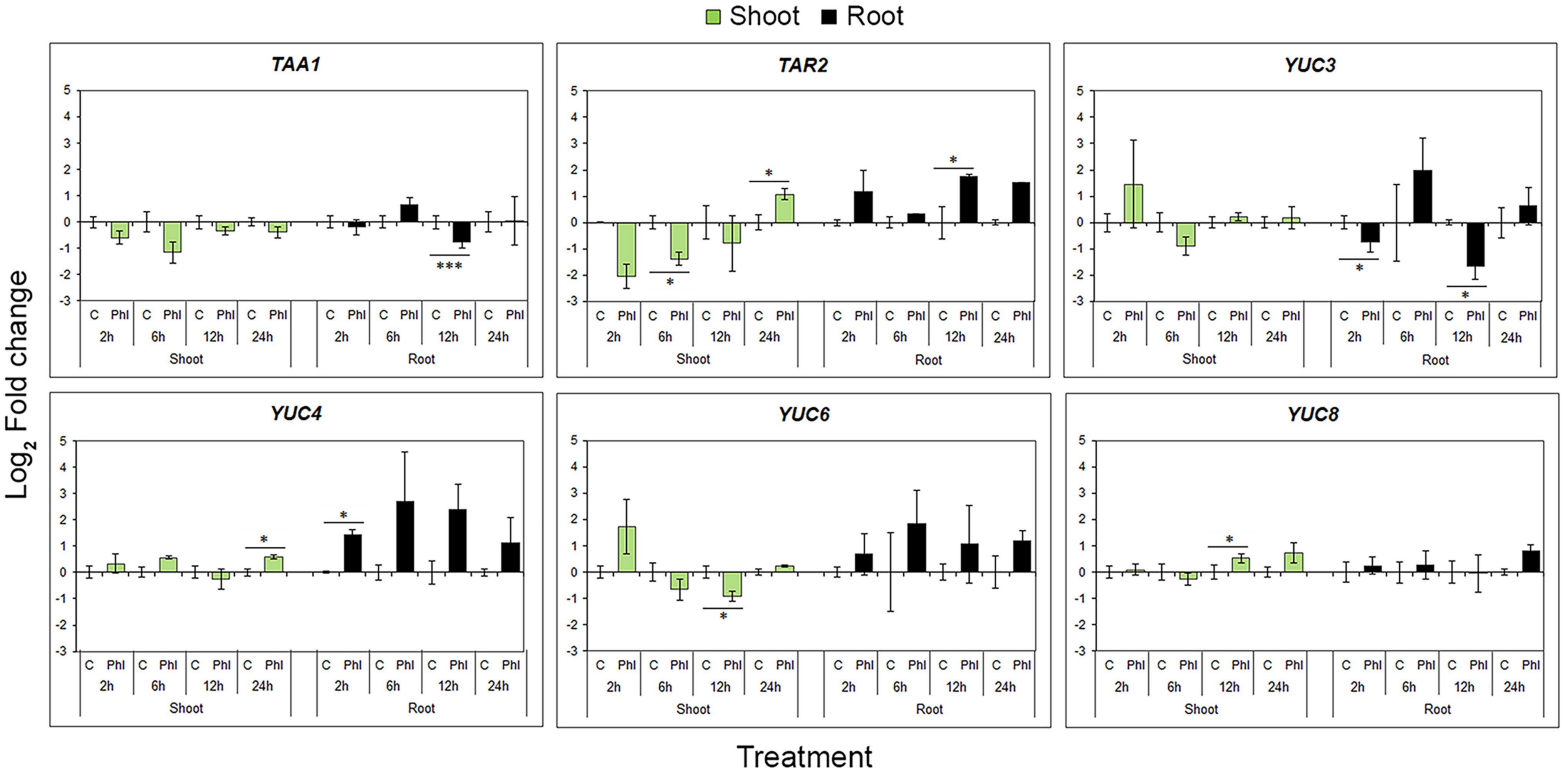
Effect of phloretin on the expression of genes involved in the auxin IPyA biosynthetic pathway in Arabidopsis. Expression levels of *TAA1, TAR2, YUC3, YUC4, YUC6* and *YUC8* genes in Arabidopsis [*Arabidopsis thaliana* (L.) Heynh.] Col-0 shoots and roots at 2, 6, 12 and 24 h of treatment with 500 μM phloretin. Expression levels were calculated relative to an expression of corresponding control according to the ΔΔCt method and presented as log_2_ transformation of fold changes. Values are presented as means ±SE from three biological replicates (samples from three independent experiments). Asterisks indicate statistically significant difference at *p < 0.1 and ***p < 0.01 based on Student’s *t*-test.

In the case of biosynthetic genes, expression profiles varied or even contradicted, probably as a result of the known functional redundancy of *TAA* and *YUC* gene family members. A statistically significant decrease in *TAA1* expression in root was significant 12 h after phloretin application, while *TAR2* expression was increased at the same sample point (Figure 7). The general tendency of phloretin to up-regulate *YUCs* in roots was in contrast to the significant down-regulation of *YUC3* at 2 and 12 h (Figure 7).

In shoots, both *TAA1* and *TAR2* genes tended to decrease their expression, with the exception of *TAR2* up-regulation at 24 h of treatment. Phloretin significantly increased *YUC4* and *YUC8* expression after 24 and 12 h of phloretin treatment, respectively, but decreased *YUC6* expression at 12 h (Figure 7).

**Figure 8:**
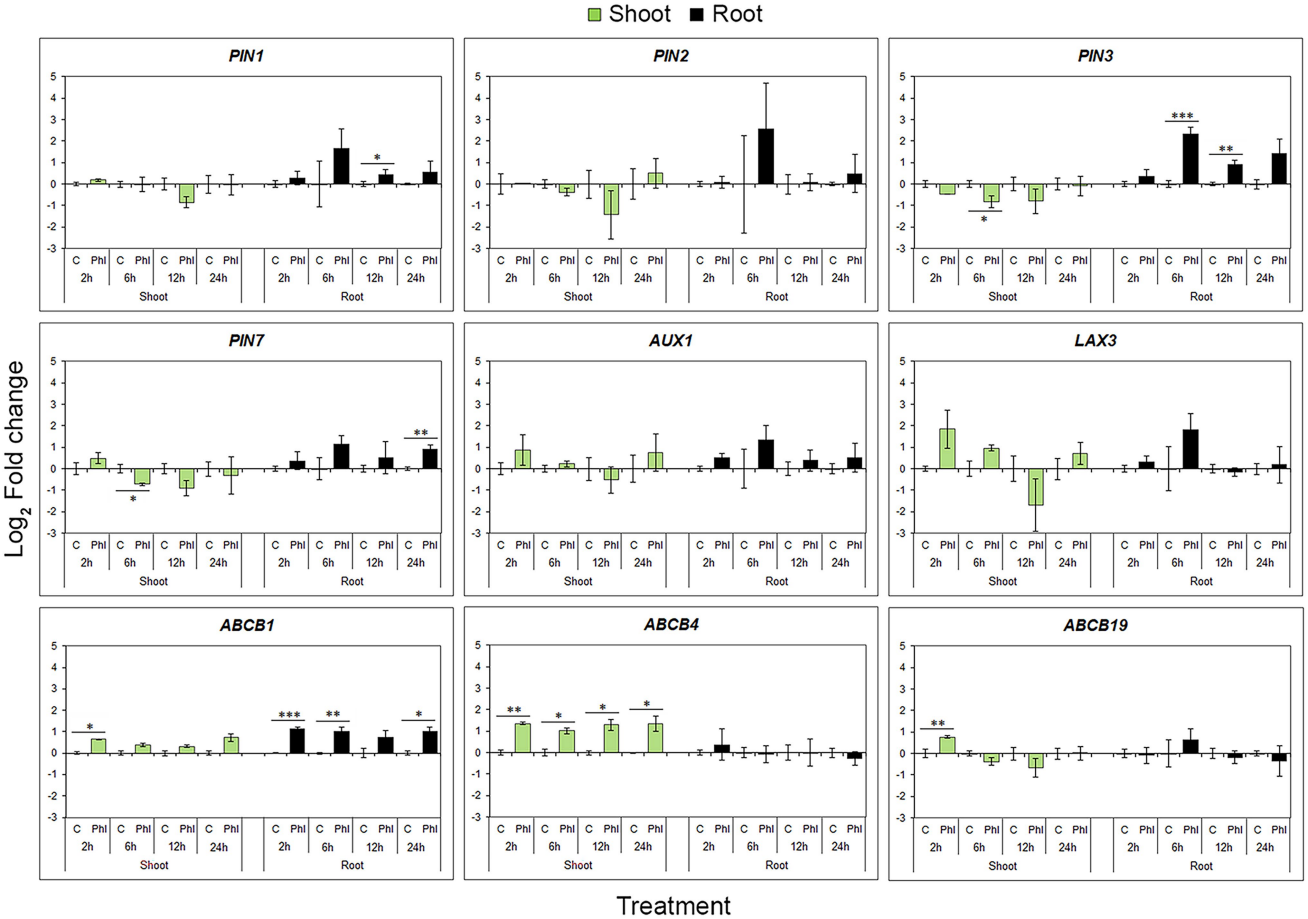
Effect of phloretin on the expression of genes involved in polar auxin transport in Arabidopsis. Expression levels of *PIN1, PIN2, PIN3, PIN7, AUX1, LAX3, ABCB1, ABCB4* and *ABCB19* genes in Arabidopsis [*Arabidopsis thaliana* (L.) Heynh.] Col-0 shoots and roots at 2, 6, 12 and 24 h of treatment with 500 μM phloretin. Expression levels were calculated relative to an expression of corresponding control according to the ΔΔCt method and presented as log_2_ transformation of fold changes. Presented data are means ± SE from three biological replicates (samples from independent experiments). Asterisks indicate statistically significant difference at *p < 0.1; **p < 0.05 and ***p < 0.01 based on Student’s *t*-test.

Unlike biosynthetic genes, phloretin coordinately affected all *PIN*s and both *AUX1* and *LAX3* genes, inducing the same time-point expression pattern represented by up-regulation at 6 h and a slight decrease at 12 and 24 h compared to the 6 h level (Figure 8). Although they show analogous time-dependent patterns of expression during phloretin treatment, only *PIN1*, *PIN3* and *PIN7* genes exhibited a statistically significant increase of expression at different time points. Unlike *ABCB4* and *ABCB19*, phloretin significantly up-regulated the *ABCB1* in roots (Figure 8).

In shoots, the patterns of gene expression within the same gene family or functional group were similar. *PIN*s expression levels showed a general tendency to decrease, but significantly only for *PIN3* and *PIN7* at 6 h of treatment (Figure 8). On the contrary, *ABCB*s have shown a tendency to increase their expression levels. The auxin influx genes (*AUX1* and *LAX3*) were not significantly affected by phloretin treatment, although they showed a tendency to react similarly (Figure 8).

In general, phloretin had the greatest effect on PAT gene expression in roots, significantly up-regulating *PIN1*, *PIN3*, *PIN7* and *ABCB1* genes in a very specific time point pattern. In shoots, phloretin elevated *ABCB* transporter genes expression, but down-regulated the expression of *PIN3* and *PIN7* genes.

### Phloretin Alters Auxin Distribution in Arabidopsis Roots

**Figure 9:**
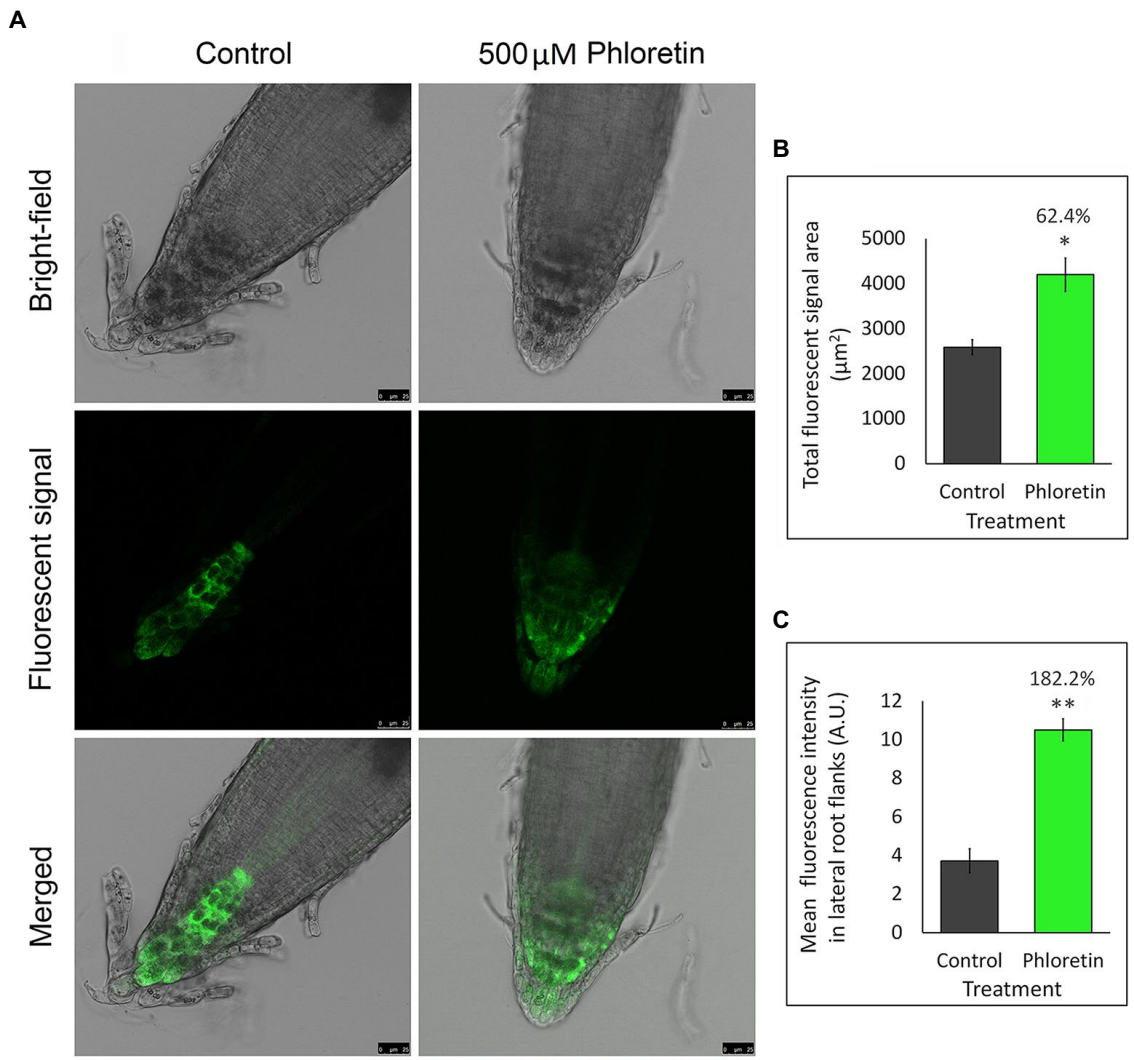
Phloretin significantly alters the distribution of auxin in Arabidopsis root tips. **(A)** Auxin distribution represented by GFP green fluorescent signal in root tips of 5 days after germination (DAG)-old DR5rev::GFP seedlings vertically grown in Petri dishes with control or 500 μM phloretin-enriched medium; Bar, 25 μm. **(B)** Quantification of the total GFP fluorescent signal area. **(C)** Mean GFP fluorescent intensity in the lateral flanks of the control and phloretin treated root tips. The lateral flanks are specified as root tip up to quiescent center, excluding the four columns of columella cell in the center of the root tip. Presented data are means ±SE of six seedlings from two independent experiments. Asterisks indicate statistically significant difference at *p < 0.05 and **p < 0.01 based Student’s *t*-test, while numbers above the bars indicate the percentage increase of total GFP fluorescent signal area **(B)** or the mean fluorescence intensity **(C)** compared to control. Arbitrary units (A.U.).

Since changes in auxin level and PAT-involved genes expression may consequently reflect on auxin distribution in the root tips, we used the Arabidopsis mutant line expressing auxin-responsive reporter DR5rev::GFP to visualize and indirectly quantify auxin redistribution in the presence of phloretin (Figure 9).

DR5rev-dependent GFP fluorescence in Arabidopsis root (Figure 9A) was significantly affected by 500 μM phloretin. In control, DR5::revGFP was predominantly expressed in columella and stem cells niche. Minimal expression was observed in the stele, and sporadically in the cells of the epidermis and lateral root cap. Phloretin treatment significantly increased the total GFP fluorescence area which expanded to the entire region of the columella and lateral root cap, including some epidermal and cortex cells of the root tip (Figures 9A,B). The mean fluorescence intensity measured in lateral flanks of the root apex was 2.8-fold higher in phloretin-treated seedlings compared to the control (Figure 9C). The obtained results indicated a significantly altered auxin distribution and its accumulation in the lateral parts of the root apex, especially in the lateral root cap and epidermis and cortical cells which are the main paths of basipetal auxin flow in the roots.

## Discussion

Phloretin is a plant-based secondary metabolite specific to apple species known for its human health-promoting properties (Behzad et al., 2017). For the first time, we characterized phloretin as an allelochemical that exhibited harmful effects on plant species outside the genus *Malus*. When tested on Arabidopsis, significant inhibition of the growth of primary and lateral roots and leaves were observed, as well as severe morphological abnormalities and agravitropic behavior of seedlings. Histological examination revealed a serious disturbance in the root architecture, which resulted in meristematic and elongation zone length reduction. Similar consequences of phytotoxic activities have been also found in other plant allelochemicals (Sánchez-Moreiras et al., 2008; Soltys et al., 2012; Zhang et al., 2018). However, phloretin induced changes including stunted growth, reduced root system with brownish root color and epidermal cells separating from the subjacent cell layers, resemble a lot to the phenotype of ARD affected apple trees (Yim et al., 2013; Winkelmann et al., 2019). Although it is suggested that many factors are involved in the etiology of ARD (Winkelmann et al., 2019), it is likely that the role of phloretin in the development of ARD symptoms is not negligible.

The inhibitory effect of phytotoxic allelochemicals on roots has been widely reported as a result of changes in the balance of plant hormones, where auxin is mainly responsible for such action. Thus, the accumulation of auxin in the epidermis of the distal elongation zone inhibited elongation of the root and shortened cells in Arabidopsis roots exposed to salicylic acid (Pasternak et al., 2019). Similarly, increased auxin content reduced both meristematic and elongation zones in benzoic acid-treated seedlings (Zhang et al., 2018). Exogenously applied IAA at concentrations above 100 nM induced dose-dependent inhibition of primary root growth in Arabidopsis seedlings (Yang et al., 2021), while both 1-naphthaleneacetic acid (NAA) and 2,4-dichlorophenoxyacetic acid (2,4-D) reduced root meristem size and primary root growth at concentrations higher than 50 and 20 nM, respectively (Růzǐčka et al., 2009). Taking into account the role of auxin in cell cycle modulation and elongation, we tried to connect the retardation of meristematic and elongation zones of roots in phloretin-treated seedlings with disturbance of auxin levels. The results of auxin metabolome profiling confirmed our hypothesis which indicated a more than threefold increase in the IAA content in Arabidopsis roots shortly after phloretin application.

Some studies have reported that environmental factors, such as salt and osmotic stresses, affect not only IAA levels, but even more the quantities of its precursors, conjugates and degradation products (Pavlović et al., 2018; Smolko et al., 2021). Our results indicated a decrease in the content of all auxin precursors except IAM in the root and particularly pronounced reduction in IAN levels shortly after phloretin treatment. IAN is not exclusively involved in auxin biosynthesis in Arabidopsis, as nitrilases play a prevalent role in glucosinolate metabolism and processes such as cyanide detoxification and camalexin homeostasis (Vorwerk et al., 2001; Su et al., 2011). Since camalexin is the major phytoalexin that accumulates after infections (Tsuji et al., 1992; Thomma et al., 1999; Glawischnig, 2007) or abiotic stresses (Zhao et al., 1998; Bouizgarne et al., 2006; Kishimoto et al., 2006), it is possible that a significant decrease in IAN levels in roots during phloretin treatment is a consequence of its depletion in intensive camalexin biosynthesis rather than in IAA overproduction.

Maintenance of auxin homeostasis is of the utmost importance for plants and is regulated by the balance among three processes: auxin storage, reactivation and irreversible deactivation. Recent study on auxin metabolism suggests that these processes are part of GH3-ILR1-DAO (amidosynthetases-IAA-Leu-Resistant - DIOXYGENASE FOR AUXIN OXIDATION 1)-mediated metabolic pathway. IAA conjugates IAA-Asp and IAA-Glu are the key metabolites and can serve both as reversible storage forms and as degradative forms that are irreversibly oxidized by DAO to form oxIAA-Asp and oxIAA-Glu, which are then hydrolyzed to oxIAA. The balance between recycling and irreversible deactivation of auxin could be turned towards the second process by IAA over-production *in planta* or its exogenous application. Our auxin metabolome profiling results indicated that the content of oxIAA in the roots of Arabidopsis was significantly elevated and that it closely followed the increase in IAA levels shortly after phloretin treatment. Apparently, the increase in IAA levels in roots led to activation of the oxIAA catabolic pathway as an attempt to eliminate excessive auxin and maintain auxin homeostasis. However, quantity of IAA-Asp and IAA-Glu was not significantly changed or was under control levels in phloretin-treated seedlings. The results of Hayashi et al. (2021) indicated that AtDAO1 enzyme prefers IAA-amino acids to IAA since its oxidation activity for IAA-Glu was c. 6 × 10^5^ times higher than for IAA in an *in vitro* assay. Thus, excessive accumulation of IAA presumably led to the production of oxIAA through the rapid conversion of IAA-Asp and IAA-Glu in their oxidized forms shortly after phloretin treatment. In addition, the higher Km value of AtDAO1 for IAA-Asp than for IAA-Glu reported in Hayashi et al. (2021) could explain why IAA-Glu content was more reduced than IAA-Asp under phloretin treatment.

As local biosynthesis and transport of auxin act in concert to establish and maintain auxin morphogenic gradients in roots (Brumos et al., 2018), we evaluated the expression of key genes involved in the IPyA biosynthetic route and PAT in Arabidopsis. Despite the general tendency of phloretin to up-regulate auxin biosynthetic genes in roots, the level of *TAA1* transcript was significantly reduced in roots after 12 h of phloretin treatment, at the same time point when transcription of *TAR2* was elevated. This is presumably the result of the shared role of *TAA1* and *TAR2* in the regulation of auxin maximum formation in meristematic root tissue due to the known functional redundancy of TAA1 and TAR2 and overlapping expression patterns in young seedlings (Stepanova et al., 2008). Similarly, the expression of *YUC3* in roots was antagonistic to *YUC4*, while *YUC6* and *YUC8* remained unchanged. Exploring expression profiles of cucumbers’ *YUC* genes under different stress treatments, Yan et al. (2016) reported that expression of *CsYUC10b* was dramatically increased, while *CsYUC4* was repressed in response to low temperature. Similarly, *CsYUC10a* and *CsYUC11* acted against up-regulation of *CsYUC10b* under salinity stress, suggesting that distinct YUC members antagonize each other under the same type of stress to maintain appropriate auxin levels. Actually, the fact that enzymes catalyzing both steps of the IPyA biosynthetic route are encoded by multigenic gene families offers a simple mechanism for generating an array of different expression patterns. Thus, the phloretin-induced elevation of endogenous auxin levels is likely to be achieved by the selective expression of particular *TAA1/TAR* and *YUC* gene family member at different time points after phloretin application.

In contrast, phloretin coordinately affected all *PIN*s and both *AUX1* and *LAX3* genes inducing the same time-point pattern of expression, but significantly up−/down-regulated only *PIN1*, *PIN3* or *PIN7* in roots and shoots, respectively. PIN1 is found postembryonically in the root stele (Gälweiler et al., 1998) and it is responsible for acropetal auxin flow towards the root tip, together with another two members of PIN family of auxin transporters: PIN3 and PIN7. Both *PIN3* and *PIN7* genes show a similar pattern of expression in root pericycle and columella cells, redirecting the auxin flow downwards to the root tip and laterally towards the flanks of the root apex (Friml et al., 2002; Billou et al., 2005). Therefore, it is likely that phloretin-induced up-regulation of *PIN1*, *PIN3* and *PIN7* genes complements local auxin biosynthesis generating more robust auxin maxima in root tips and induces increased auxin accumulation in lateral parts of the root tips, as visualized in Arabidopsis transgenic line DR5rev::GFP. The phytotoxicity of bisphenol A (Bahmani et al., 2020) and weisiensin B (Li et al., 2019) was also based on the increased expression of *PIN1, PIN3* or *PIN7* in roots.

Treatment with phloretin also induced a strong agravitropic phenotype of Arabidopsis seedlings. As the lack of starch in the starchless mutants (Caspar et al., 1989; Kiss et al., 1989) or physical elimination of columella cells (Blancaflor et al., 1998; Tsugeki and Fedoroff, 1999) led to severely attenuated gravity responses, redistribution of starch-filled amyloplasts can be considered essential for plant gravity sensing (Baldwin et al., 2013). Light microscopy examination of the root tips of phloretin-treated Arabidopsis seedlings confirmed the assumption that reduction of starch content in columella cells was in the bases of agravitropic phenotype of treated seedlings. Similarly, the allelochemicals narciclasine and artemisinin inhibited the root gravity sensing as a result of reduced starch levels in the root tips of Arabidopsis (Na et al., 2011; Yan et al., 2018).

The normal gravitropic response further depends on the regular flow of auxin in roots mediated by the symmetrical positioned PIN3 which enables uniform auxin flow toward the flanks of the root tip from where it enters the basipetal stream depending on PIN2 (Friml et al., 2002; Kleine-Vehn et al., 2010). Some polyphenols such as ellagic acid and cold/heat stress are able to disturb the gravitropic root response by affecting the expression, distribution or trafficking of PIN2 and/or PIN3 proteins (Shibasaki et al., 2009; Hanzawa et al., 2013; Yan et al., 2015). Presumably, phloretin-induced up-regulation of *PIN3* together with reduced starch content in gravity sensing columella cells are the main factors responsible for agravitropic growth and morphological abnormalities of treated Arabidopsis seedlings.

The activity of the proteins from the ATP-BINDING CASSETTE transporter superfamily, especially ABCB1, ABCB4, and ABCB19, contributes to the directed movement of auxins in plants (Geisler et al., 2005; Terasaka et al., 2005; Lewis et al., 2007). However, the strict role of the ABCBs remains controversial (Geisler et al., 2017). ABCB19 is localized in the vascular tissues of the hypocotyl and root, contributing to root-ward transport of auxin, while ABCB1 and ABCB4 are involved in the loading and transport of auxin into the shoot-ward stream from the root apex. Accordingly, mutants deficient in ABCB1 protein display reduced shoot-ward auxin transport (Geisler et al., 2005). Since ABCB transporters stably retain their localization in plasma membranes in response to internal and external cues (Cho et al., 2012), changes in their expression levels, such as the observed increase in expression of *ABCB*s in shoots and *ABCB1* in roots, could be a way of modulating the root-ward and shoot-ward auxin transport, respectively, contributing to the *PIN*s´ driven auxin accumulation in roots and agravitropic phenotype of phloretin-treated seedlings. In addition, increased expression of *ABCB1* in phloretin-treated roots at time points coinciding with elevated levels of IAA and oxIAA, suggests that ABCB1 could mediate transport of IAA breakdown products in the regions exposed to high auxin concentrations, as also proposed by Geisler et al. (2005).

Phloretin human health-promoting properties (Behzad et al., 2017) in combination with its phytotoxic effects could be essential in development of new prospective bioherbicidal agent. Since phloretin could be obtained from waste products such as apple tree residues (fallen apple tree leaves) and juice industry by-product (apple pomace) by deglycosylation of phlorizin, it makes phloretin-based bioherbicides a prospective economical and eco-friendly solution for weed control. In addition, grinded apple tree leaves could be also applied by mulching, since phlorizin from apple mulch is degradable by soil actinomycetes (Huang et al., 2013b) to produce phloretin.

## Conclusion

The presented study introduces the apple dihydrochalcone phloretin as a new allelopathic compound with harmful effects on species outside the genus *Malus* and provides the very first insight into the basic mechanism of its phytotoxic action. Phloretin treatment induced a significant dose-dependent growth retardation and agravitropic phenotype in Arabidopsis seedlings. Profoundly disturbed auxin metabolome profile in roots with highly increased content of IAA accumulated in the lateral parts of the root apex, accompanied with changes in the expression of auxin biosynthetic and transport genes, especially *PIN1, PIN3, PIN7* and *ABCB1*, confirms that the disturbance of auxin homeostasis is the basis of phloretin phytotoxicity. This mechanism makes phloretin an attractive candidate for a new prospective bioherbicidal compound. Insight into the physiological basis of phloretin action also paves the way for further research of phloretin role in ARD and its physiological functioning *in planta* for a better understanding of its role as a potential growth modulator in apple.

## Data Availability Statement

The raw data supporting the conclusions of this article will be made available by the authors, without undue reservation.

## Author Contributions

MS, NB, and SN designed the study. MS, DS, and MT performed morphological tests. DĆ and DS conducted the light microscopy experiments. AP performed auxin metabolite profiling analyses. MS, JS, and TĆ performed qRT-PCR analyses. MB, MS, and DS conducted confocal microscopy analyses. MS, DS, and JS analyzed the data and prepared the figures and tables. MS and DS wrote the manuscript. NB and SN provided critical editing of the manuscript. All authors contributed to the article and approved the submitted version.

## Funding

This work was supported by the Ministry of Education, Science and Technological Development of the Republic of Serbia, contract number 451-03-68/2022-14/200007.

## Conflict of Interest

The authors declare that the research was conducted in the absence of any commercial or financial relationships that could be construed as a potential conflict of interest.

## Publisher’s Note

All claims expressed in this article are solely those of the authors and do not necessarily represent those of their affiliated organizations, or those of the publisher, the editors and the reviewers. Any product that may be evaluated in this article, or claim that may be made by its manufacturer, is not guaranteed or endorsed by the publisher.

## Supplementary Material

The Supplementary Material for this article can be found online at: https://www.frontiersin.org/articles/10.3389/fpls.2022.875528/full#supplementary-material
