# Supplementary material for: New Insights Into the Activity of Apple Dihydrochalcone Phloretin: Disturbance of Auxin Homeostasis as Physiological Basis of Phloretin Phytotoxic Action
Source: Front Plant Sci. 2022 Jul 7;13:875528. doi: 10.3389/fpls.2022.875528 (PMC9302884; doi:10.3389/fpls.2022.875528)
Supplement: Supplementary file 1 [file Table_1.DOCX]

**Table S1** List of primers used for qRT-PCR analyses of genes involved in auxin biosynthesis and polar auxin transport in Arabidopsis (*Arabidopsis thaliana* (L.) Heynh.) Col-0

| **Gene name** | **GenBank™ accession number** | **Sequence (5'→3')** | **Amplified product size (bp)** | |
| --- | --- | --- | --- | --- |
| *TAA1* | NM_105724.3 | F: ATCTTACCCTGCGTTTGCGT  R: GCTGACTCGGACATGCTTCT | | 130 |
| *TAR2* | NM_202879.2 | F: TCGTTTCCAAGGTCTGCCAT  R: GCCACAGTGAAAGGATCGCTA | | 194 |
| *YUC3* | NM_100340.4 | F: ATTTTGGCGGCGATGTTCTC  R: TGGGTTTGCTCCGTGATTGT | | 137 |
| *YUC4* | NM_121170.3 | F: TAACGAGGAACGGGGCAAAG  R: CGGCGTTTTTGGCATTCCT | | 143 |
| *YUC6* | NM_122473.3 | F: AGGGTTCTTGTCGTCGGATG  R: TGGGCAGCCATTTCAGTAAGA | | 172 |
| *YUC8* | NM_119016.3 | F: ATCAACCCTAAGTTCAACGAGTG  R: GAAAACTCAGAAAGACCATCAATC | | 193 |
| *PIN1* | NM_106017.3 | F: TCAAGGCTTATCTGCGACAC  R: AGTTAGAGTTCCGACCACCA | | 155 |
| *PIN2* | NM_125091.3 | F: TCTTGTTTGAGTTCCGTGGG  R: TTCCGTCGTCTCCTATCTCC | | 153 |
| *PIN3* | NM_105762.2 | F: ATTGTGCCCTTTGTGTTTGC  R: TAAACCAGCGTGATCGGAAG | | 104 |
| *PIN7* | NM_001084115.1 | F: AAATGCTGGTCCGATGAACG  R: CTGCTGTGGAGTTACACCGA | | 104 |
| *AUX1* | NM_129368.2 | F: GTTGAATGCTTTCGTGGTGG  R: CGATCTCTCAAAGACGGTGG | | 188 |
| *LAX3* | NM_106418.3 | F: CTTACCTTTGCTCCTGCTCC  R: GACCATACTTGCCCATCCTC | | 153 |
| *ABCB1* | NM_129247.2 | F: AGATCATGCTGCTTGACGAG  R: AGCCTATGAGCCACGACTAT | | 118 |
| *ABCB4* | NM_130268.3 | F: GTGCTCTTGACGCTGAGTCT  R: CTTTCTCAACGATGACGCCG | | 153 |
| *ABCB19* | NM_113807.2 | F: ACCCGTTTGAGCCATTCACT  R: TTCAGGGGCACGAGTCTTTC | | 150 |
| *ACT7* | NM_121018.4 | F: ACAGGAAATGCTTCTAAGTGTGTCT  R: ACACAAGACTTCTTGACACAACCA | | 171 |

*ABCB* (*ATP-BINDING CASSETTE-B*), *ACT7* (*ACTIN 7*), *AUX1* (*AUXIN RESISTANT 1*), *LAX3* (*AUXIN TRANSPORTER-LIKE PROTEIN 3*), *PIN (PIN-FORMED),* *TAA1* (*TRYPTOPHAN AMINOTRANSFERASE OF ARABIDOPSIS 1*), *TAR2* (*TRYPTOPHAN AMINOTRANSFERASE RELATED 2*), *YUC* (*FLAVIN-BINDING MONOOXYGENASE*)
